# Phylogenomics Reveals Deep Divergences and Cryptic Species Within a Rare Sand‐Dwelling Milkweed, *Asclepias tomentosa* Elliott

**DOI:** 10.1002/ece3.71942

**Published:** 2025-08-08

**Authors:** Daniel P. Duran, Jason E. Ksepka, Scott A. Davis, William Godwin, Robert A. S. Laroche

**Affiliations:** ^1^ Department of Environmental Science Rowan University Glassboro New Jersey USA; ^2^ The Milkweed Foundation Apalachicola Florida USA; ^3^ Sam Houston State Natural History Collection Huntsville Texas USA; ^4^ Department of BioSciences Rice University Houston Texas USA

**Keywords:** Apocynaceae, Asclepiadoideae, biodiversity, integrative taxonomy, new species, velvetleaf milkweed

## Abstract

Integrative taxonomy incorporates multiple data types to delineate and describe species and discovers hidden biodiversity (i.e.*,* ‘cryptic’ species) previously undetected when relying solely on morphology. Phylogenomic data, when combined with morphological and ecological data, have revealed many cryptic species in animal and plant taxa with large geographic ranges and disjunct distributions. In the present study, we examined the genetic structure of the rare milkweed species, *Asclepias tomentosa* Elliott, found in the southeastern USA, with disjunct sets of populations occurring in Texas, Florida, and the Carolinas. Using multiple analyses, including a phylogenomic tree, population structure analysis, principal component analysis of SNP data, calculations of F_ST_, and a Bayesian species delimitation model, we uncovered three well‐separated genetic lineages, each corresponding to the major geographic areas. Populations from Texas were most deeply separated from the rest and were differentiated in every analysis, including previously unrecognized morphological characters. Herein, we describe these Texas populations as a new species, *Asclepias tonkawae* sp. nov., and discuss the conservation implications of this discovery.

## Graphical abstract

Phylogenomic analyses demonstrated that the rare milkweed, *Asclepias tomentosa* , exhibited deep divergences correlated with geography and revealed the presence of cryptic species. A new species, A. tonkawae, sp. nov., is described from the Texas populations.

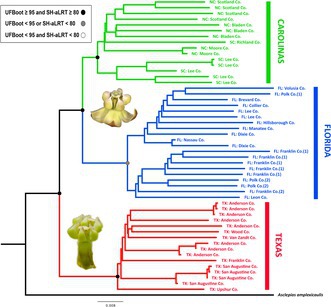

## 1 Introduction

Discovering and describing biodiversity during an age of rapid species declines is critically important and allows for a fundamental understanding of the varieties of living things on Earth (May 2011). Without knowing the accurate taxonomic and evolutionary units present in a given geographic area, scientists and policymakers cannot know what to conserve (Mace 2004). One major problem in biodiversity science is determining the number of species in any taxonomic group. “Cryptic species” which are species that exhibit low morphological differentiation but exhibit considerable genetic differentiation from other closely related species (e.g., Smith et al. 2006; Bickford et al. 2007; Burns et al. 2008; Janzen et al. 2017), may contribute to the problem.

For the vast majority of eukaryotic taxa, species delineation and description have been based exclusively on morphology, with a lesser reliance on ecological, behavioral, or other characters (Dayrat 2005); this is implicitly based on the concept that fixed morphological differences in two or more sets of populations are the result of the splitting of gene pools from a single ancestral species. Recognizing species as entities that are distinct with respect to physical structures is known as the Morphological Species Concept (MSC; Cronquist 1978) and is also a component of other related species concepts (Coyne and Orr 2004; Wilkins 2009). In more recent studies, taxonomists have incorporated molecular data into taxonomic revisions and species descriptions (e.g., Brandão‐Dias et al. 2022; Duran et al. 2024), resulting in substantial changes to established taxonomic frameworks that are at odds with the MSC (e.g., Barrowclough et al. 2016; Ruiz‐Garcia et al. 2018; Titus et al. 2019; Kim et al. 2022; Laroche et al. 2023). Since the early 2000s, there has been significantly more reliance on primarily or exclusively molecular data, especially the use of mitochondrial DNA for “DNA Barcoding” (Hebert et al. 2003), mostly used in animal taxa. This rapid change in taxonomic practice presented a challenge for the taxonomic community, especially when taxonomic decisions were made using a single marker such as mtDNA, which may be discordant with patterns inferred from genomic data due to a number of evolutionary phenomena (Funk and Omland 2003; Ballard and Whitlock 2003). Integrative taxonomy aims to incorporate multiple types of data, including traditional morphological, molecular (genomic and single‐locus genealogies), and to a lesser degree, ecological, behavioral, or other types of data (Dayrat 2005; Will et al. 2005; Pante et al. 2015; van Elst et al. 2025). Most, if not all, professional taxonomists and systematists would agree that collecting and analyzing multiple lines of evidence would lead to more robust conclusions and stable taxonomy.

The milkweed genus *Asclepias* L. *sensu stricto* contains about 130 species of predominantly herbaceous perennial flowering plants distributed throughout the western Hemisphere, with the bulk of the diversity found in southwestern North America (Woodson 1954). These plants are well studied with respect to their systematics (Fishbein et al. 2011; Fishbein et al. 2018), evolutionary defenses against herbivores (e.g., Agrawal and Fishbein 2008; Agrawal and Weber 2015) pollinator interactions (e.g., Kephart and Theiss 2004; Theiss et al. 2007; Burger et al. 2024) and ecological importance (e.g., Morse 1985; Pleasants and Oberhauser 2012; Pocius et al. 2017). As with many taxonomic groups, the majority of species were described in the 18th and 19th centuries, with a small fraction of the diversity described after the beginning of the 20th century. To date, species have been described based on floral and vegetative traits. The potential for cryptic species is high in the genus *Asclepias*, with some nominal species containing large ranges, named subspecies or forms, disjunct distributions, or ecological differences in parts of their ranges.

**FIGURE 1:**
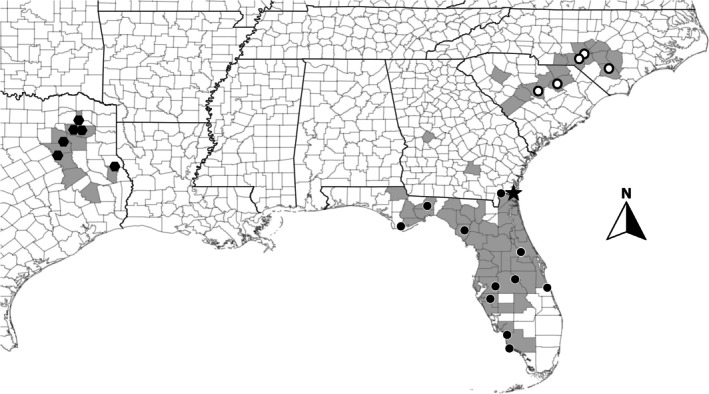
Distribution of *Asclepias tomentosa* . Shaded counties represent known records for the species from the USDA Plants Database and iNaturalist. Genomically sampled areas are as follows: Black circles represent sampled populations that belong to the Florida clade (see Figure 2), open circles represent sampled populations that belong to the Carolinas clade, and black hexagons represent sampled populations that belong to the Texas clade. The black star represents the type locality for the species (Elliott 1817).

**FIGURE 2:**
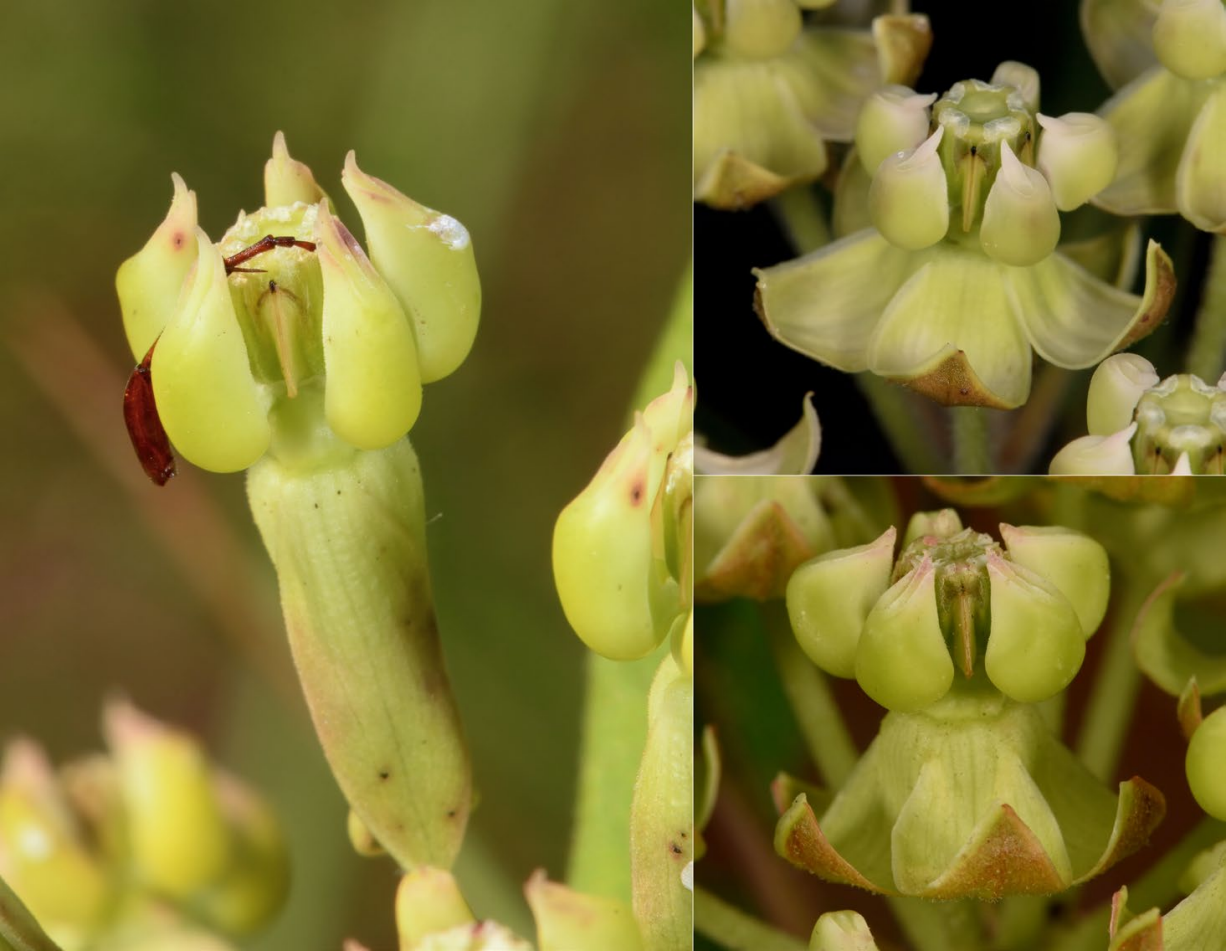
Left: An individual from Anderson County, Texas, with a corolla strongly reflexed and appressed or nearly appressed to the pedicel. Top right: An individual from Franklin County, Florida, exhibiting representative floral morphology with corolla partly reflexed and tips antrorse. Bottom right: An individual from Scotland County, North Carolina, with corolla partly reflexed and tips antrorse.

The velvetleaf milkweed, *Asclepias tomentosa* Elliott, is a species with an interesting distribution in sandy regions of the southeastern United States (Figure 1). It is apparently disjunct in multiple places (Sorrie 2016), being found throughout the Sandhills region of the Carolinas along with a single record from central western Georgia (also part of the Sandhills), as well as throughout the panhandle and peninsular Florida and southern Georgia. Nearly 1000 km separate the closest populations of the Florida panhandle from those of eastern Texas. In Texas, the species is only distributed in the loose, sandy soils of the Carrizo and Sparta formations. Although the species has a widespread distribution, it is uncommon to rare throughout all of its range (Sorrie 2016; NatureServe Explorer 2025). Morphologically, the species is characterized by a dense fine pubescence and sessile or subsessile inflorescences with yellowish to greenish flowers, occasionally suffused with purple (Woodson 1954). Although all populations key to *A. tomentosa* in Woodson (1954), the second author observed previously undocumented differences in floral morphology between the populations in Texas and all others (Figure 2).

Based on observed geographic disjunction and newly discovered morphological differences, we hypothesized that cryptic species may exist within nominal *A. tomentosa* . We tested this hypothesis using multilocus genomic data, and our results indicate substantial genetic subdivisions into three major, reciprocally monophyletic lineages, each corresponding to different parts of the geographic distribution. Additional population genomic analyses all support the existence of deep evolutionary separation and cryptic species. Future studies may reveal even more cryptic diversity within the North American milkweeds and highlight previously unseen conservation priorities.

## 2 Materials and Methods

### 2.1 Population Sampling

Historic localities for *A. tomentosa* were obtained from published records and publicly available online data sources, including USDA Plants Database, iNaturalist, and the Southeast Regional Network of Expertise and Collections (SERNEC). Collecting efforts were conducted to include as many geographic areas as possible (Figure 1). All specimens and their collection localities are indicated in Table S1. Five localities were sampled from the Carolinas; 11 were sampled from Florida, and six were sampled from Texas. Despite our efforts, we were unable to obtain samples from the historic Coffee County and Taylor County, Georgia localities. Most populations consisted of one or a few individuals. We sampled a single leaf from each plant, ranging from one to 10 individuals per population, based on what was available at each site. In total, 83 plants were sampled, including one outgroup taxon, *Asclepias amplexicaulis* . Leaf material was rapidly desiccated in silica gel. Collection permits were obtained from all relevant governmental agencies and parks (see Acknowledgements) and are available upon request.

### 2.2 Multilocus Marker Generation and 2b‐RAD Analysis

We used a 2b‐RAD procedure (Wang et al. 2012) to produce reduced representation libraries and generate an SNP dataset for all analyses. Genomic DNA was extracted from dried leaf tissue using a CTAB method (Aboul‐Maaty and Oraby 2019). Approximately 500 ng of genomic DNA was used for enzyme optimization, digestion, library preparation, and sequencing (all protocols can be found at https://www.cd‐genomics.com/ddrad‐seq.html). Following enzyme optimization, samples were digested using the BsaXI restriction enzyme and paired‐end sequenced on an Illumina Hiseq Xten/NovaSeq platform with 150 bp read lengths.

The paired‐end reads were merged using PEAR software (Version 0.9.6) (Zhang et al. 2014). The terminal 3‐bp positions were also excluded from each read to eliminate artifacts that might have arisen from ligation sites. Reads with ambiguous bases (N) that exceed 8%, poor quality (15% nucleotide positions with a Phred quality < 30) or those without restriction sites were removed. De novo genotyping was performed using RADTYPING (Fu et al. 2013) with default parameters. Initially, all sequences of a few representative samples were pooled together to assemble into exactly matching read clusters (i.e., representing individual alleles), and then “allele” clusters were further merged into “locus” clusters by allowing two mismatches using USTACKS (Catchen et al. 2013). A collection of consensus sequences from all of the “locus” clusters comprises a set of representative reference sites. High‐quality reads of each individual were aligned to the constructed reference using SOAP2 (Li et al. 2009) (version 2.21), and then the ML algorithm was performed to determine the most likely genotype. In order to obtain robust results in the subsequent analyses, the following criteria were applied for SNP filtering: (1) Segregating markers that could be genotyped in at least 80% of the individuals were kept for analyses; (2) SNPs with a minor allele frequency (MAF) < 0.01 were discarded; (3) Polymorphic loci with more than two alleles possibly derived from sequencing or clustering errors were excluded; (4) Tags with more than two SNPs were excluded. For genetic analysis, each genotype with markers was assembled head to tail, and missing sites were replaced by “–.” In total, after all filtering steps, there were a total of 2417 SNPs that were used in all analyses.

### 2.3 Multilocus Nuclear Trees

Phylogenetic relationships were inferred using IQ‐TREE v. 1.6.9 (Nguyen et al. 2015). Model selection was performed using ModelFinder in IQ‐TREE by specifying the command ‐MFP + merge with the best model chosen using BIC (Kalyaanamoorthy et al. 2017). The tree with the best maximum‐likelihood score was selected from 200 independent searches. For each of the 200 runs, we estimated nodal support using 1000 ultrafast bootstraps and 1000 SH‐aLRT tests. We used the ‐bnni command to avoid severe model violation resulting in overestimation of nodal support when performing ultrafast bootstraps. All other parameters were left as default. Trees were visualized with FigTree v. 1.4.4 (http://tree.bio.ed.ac.uk/software/figtree).

### 2.4 Species Delimitation Model

The BEAST (Version 2.7.3) package SPEEDEMON (Version 1.1.0) was used as a further test of species delimitation (Douglas and Bouckaert 2022) on the ingroup taxa of nominal *A. tomentosa* populations. SPEEDEMON's Yule Skyline Collapse model was employed, where samples with an estimated ancestral species time below a threshold, epsilon, are collapsed into a single species. Epsilon values of 10^−3^ and 10^−4^ were tested with an MCMC chain of ten million and all other model parameters left as default. These epsilon values were within the range of values found to be effective in Douglas and Bouckaert's (2022) paper and successfully predicted species boundaries in other delimitation studies (e.g., Laroche et al. 2023; Duran et al. 2024).

### 2.5 Population Structure Analysis

We used ADMIXTURE v. 1.3.0 to perform unsupervised assignment of individuals to K populations using a maximum‐likelihood clustering algorithm (Alexander et al. 2009). The model identifies population assignment of individuals based on patterns of Hardy–Weinberg and linkage disequilibrium. Values of *K* = 1 to 10 were run with 10 different seeds for 10 repetitive analyses. Optimal *K* was determined by the CV (cross‐validation error), and the smallest CV is best supported as the optimal value. *K* = 2 was a direct test of the taxonomic hypothesis that Texas populations were separate from all eastern populations; *K* = 3 was a direct test of the hypothesis that there were three distinct evolutionary units: those from Texas, Florida, and the Carolina Sandhills.

### 2.6 Principal Component Analysis

Principal component analysis (PCA) is a multivariate statistical method that examines the correlation between multiple variables. PCA of the SNP dataset was performed using PLINK2 v.2.0 (Purcell et al. 2007). We plotted the first two principal components.

### 2.7 Genetic Differentiation Estimation

Measures of genetic differentiation within and between populations were calculated using the R‐package GENEPOP (Version 1.0.5) (Rousset 2008). We calculated the genetic differentiation coefficient (*F*_ST_) and the Reynolds' genetic distance (DR), a method for estimating population divergence that is commonly used with SNP data. Other population genetic metrics included *π* (nucleotide diversity), Na (observed number of alleles), Ne (effective number of alleles), He (expected heterozygosity), Ho (observed heterozygosity), and PIC (polymorphism information content).

## 3 Results

### 3.1 Phylogeny From Genome‐Wide SNP Data

**FIGURE 3:**
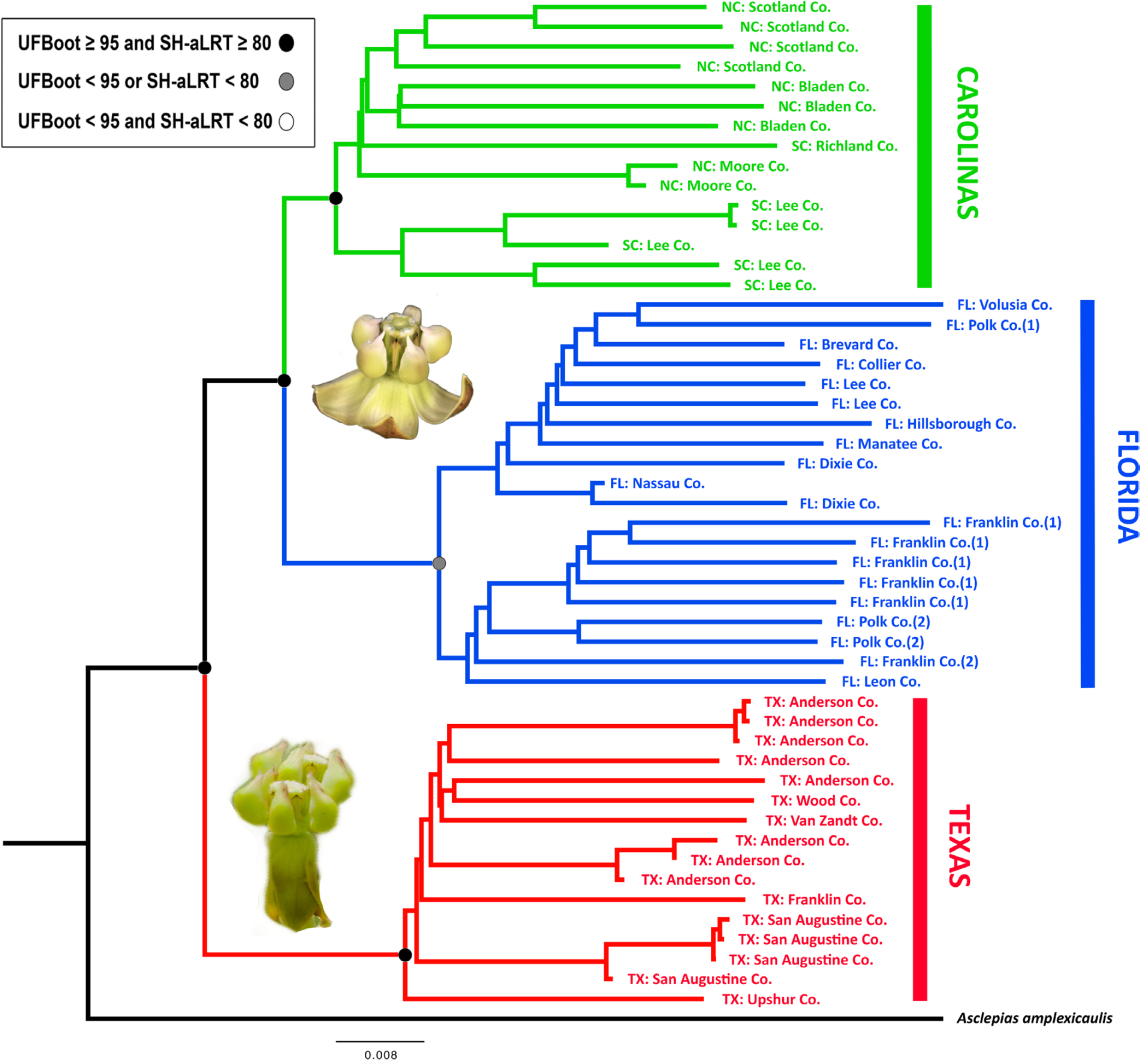
Maximum‐likelihood phylogeny inferred in IQ‐TREE based on 2417 SNPs. Individual plants are labeled with their geographic locations, indicated by a two‐letter abbreviation for state followed by the county. Exact locality data is available in Table S1. The three major clades (Texas, Carolinas, and Florida) were identified as being separate species in the Douglas and Bouckaert (2022) species delimitation model, with the Texas clade being the most divergent.

The maximum‐likelihood tree recovered clades that were congruent with the geographic subdivisions for the nominal species. The deepest split was between the populations from Texas and the eastern populations found in Florida, North and South Carolina (Figure 3), with Reynold's genetic distance (DR = 0.35–0.44) (Table 1). Within the eastern clade, there was a major divide between Florida and the Carolinas (DR = 0.24). Some additional minor geographic structuring was evident within parts of the three major clades.

**TABLE 1:** Genetic differentiation between geographic areas, calculated in GENEPOP V1.0.5. (Rousset et al. 2008). Upper right numbers are Reynold's genetic distance (DR), lower left numbers are F_ST_ values. All F_ST_s were significant (*p* < 0.01) as indicated by **.

	Texas	Carolinas	Florida
Texas	—	0.350	0.441
Carolinas	0.296**	—	0.238
Florida	0.357**	0.212**	—

### 3.2 Species Delimitation Model

SPEEDEMON identified only a single species topology within the 95% credible set. The identified topology had 100.0% posterior support, and these results were robust to changes in epsilon threshold values. This topology included members of the Texas Clade, Florida Clade, and Carolinas Clade as three distinct taxa, with the Florida Clade and Carolinas Clade being closest related to each other, in agreement with other genomic analyses.

### 3.3 Population Structure Analysis

**FIGURE 4:**
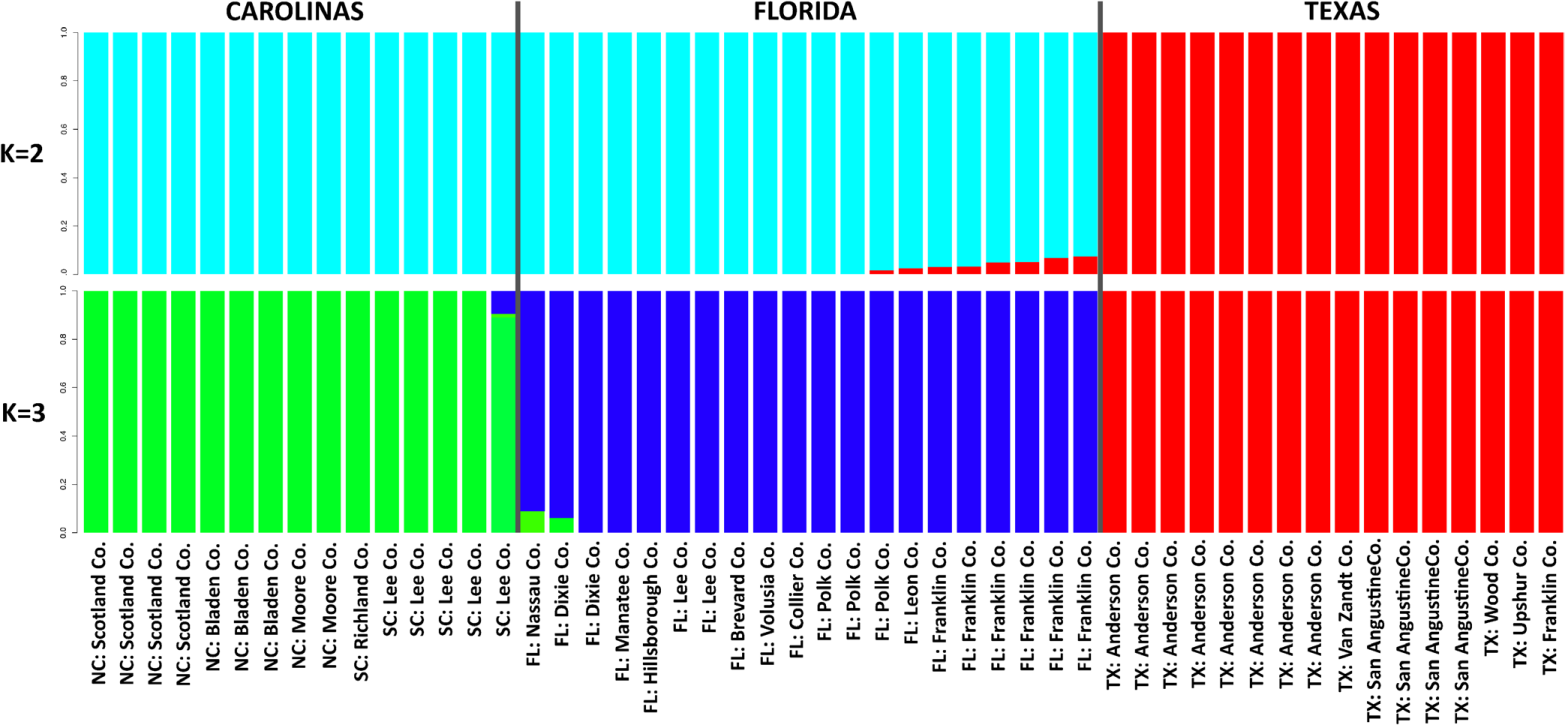
Population structure analysis implemented using ADMIXTURE (Version 1.3.0) to perform unsupervised assignment of individuals to *K* populations using a maximum‐likelihood clustering algorithm (Alexander et al. 2009). Shown are *K* = 2 (top) and *K* = 3 (bottom). Using the CV (cross‐validation error) the smallest value of *K* = 3 is best supported as the optimal value.

ADMIXTURE recovered groupings that were congruent with those observed in the phylogeny. At *K* = 2, the first split was between Texas populations and all others (Figure 4). A small (< 0.1) fraction of the Texas genome was found in some of the Florida plants, mostly in those from the panhandle. At *K* = 3, populations split into three sets: those from Texas, Florida, and the Carolinas. As with *K* = 2, there was a small amount of admixture in peripheral populations. Optimal *K* was determined to be *K* = 3, based on the cross‐validation error (CV) (Alexander et al. 2009) (Figure S1). At all additional K, the major identified groups did not change significantly.

### 3.4 PCA

**FIGURE 5:**
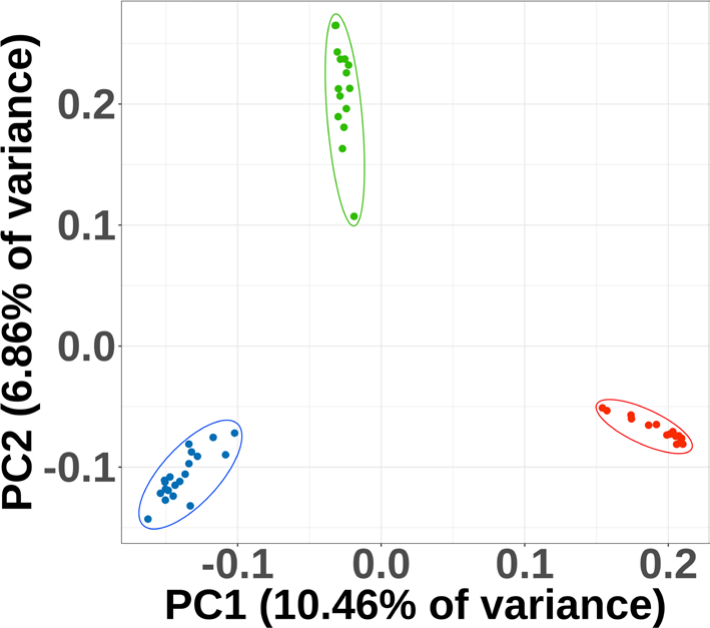
Principal component analyses (PCA) performed using PLINK2 (Version 2.0) (Purcell et al. 2007). Red points on the lower right‐hand side of the graph correspond to individuals from Texas, blue points on the lower left‐side of the graph correspond to individuals from Florida, and green points in the upper middle of the graph correspond to individuals from the Carolinas.

The first two principal components explained 10.46% and 6.86% of the total variation in the dataset. The observed results indicated that individuals grouped into three clusters: those from Texas, those from Florida, and those from the Carolinas (Figure 5). On PC1, geographic structuring was evident, and the Texas populations clustered together and were furthest from the Florida and the Carolinas populations. On PC2, the Carolinas were furthest separated from the others.

### 3.5 Population Genetic Metrics

Based on the SNP dataset, *F*_ST_ was calculated as follows (Table 1): *F*_ST_ = 0.357 between Texas and Florida; *F*_ST_ = 0.296 between Texas and the Carolinas; *F*_ST_ = 0.212 between Florida and the Carolinas. All F_ST_s were significantly different at *p* < 0.01. Measures of population genetic diversity, including *π*, Na, He, Ho, and PIC, were all lower in Texas populations and similar between Florida and the Carolinas (Table S2).

## 4 Discussion

### 4.1 Concordance Between Molecular Analyses and Geography

There was broad concordance between all genomic analyses, and each analysis recovered groupings that corresponded to the same major geographic areas. In our maximum‐likelihood phylogeny, populations of historically *A. tomentosa* from Texas (now *A. tonkawae*, sp. nov.) were reciprocally monophyletic with respect to true *A. tomentosa* from Florida and the Carolinas, each of which was also reciprocally monophyletic (Figure 3). Reciprocal monophyly has been used as a criterion for delimiting evolutionarily significant units (ESUs) (Moritz 1994) and species (de Queiroz 2007), and reciprocal monophyly may require significant evolutionary time (Hudson and Coyne 2002); conversely, its absence typically reflects more recent divergence.

Species delimitation methods, based on the Multispecies Coalescent (MSC) model (e.g., Yang and Rannala 2010; Bouckaert et al. 2019; Douglas and Bouckaert 2022), do not require reciprocal monophyly to extrapolate species boundaries. Therefore, this type of analysis may detect distinct taxa that have speciated more recently. These methods are known to have limitations when geographically widespread species are undersampled (e.g., Chambers and Hillis 2020). Given our representative geographic sampling (Figure 1), this issue should not be problematic. Additionally, these methods assume no gene flow or natural selection, a more significant issue for species that are in geographic contact (Smith and Carstens 2020). All of our putative species identified using the SPEEDEMON Bayesian delimitation model (Douglas and Bouckaert 2022) are allopatric. SPEEDEMON recovered a deep split between *A. tonkawae* and the sensu stricto *A. tomentosa* and a more recent split between populations from Florida and the Carolinas.

Population‐level analyses (FST, ADMIXTURE, and PCAs) displayed genetic structuring by geography as well, with Texas populations being most differentiated. Values of *F*_ST_ range from 0 to 1, with 0 indicating no genetic differentiation and 1 indicating complete differentiation. According to Wright (1969), who devised the metric, *F*_ST_ values of 0.00–0.05 are considered “little” genetic differentiation, 0.05–0.15 is “moderate” differentiation, 0.15–0.25 is “great” differentiation, and > 0.25 is “very great” differentiation. Hartl and Clark (1997) used similar classes, and Frankham et al. (2002) regarded *F*_ST_ > 0.15 as “significant” differentiation. Comparison of F_ST_ values between geographic areas in historically nominal *A. tomentosa* was substantially higher than those of other wide‐ranging milkweed species that have been studied. *F*_ST_ in *Asclepias syriaca* across its range was 0.002–0.082 (Boyle et al. 2023); *F*_ST_ in *Asclepias speciosa* ranged from 0.001 to 0.005 (Sussman, unpublished manuscript). One study of the dwarf milkweed clade (*Asclepias eastwoodiana, Asclepias sanjuanensis, Asclepias ruthiae, and Asclepias uncialis*) showed F_ST_ levels between the four species that ranged from 0.084 to 0.134 (Riser et al. 2019), and each of these species was supported as distinct in other analyses, including STRUCTURE and ecological niche modeling. By comparison, populations of nominal *A. tomentosa* in different geographic areas differed by *F*_ST_ of 0.212 to 0.357.

PCA of genomic data can be used to infer genetic affinities between individuals and geographic populations. This method allows for fine‐scale population structure to be detected, the result of various demographic processes such as isolation, colonization, migration, and admixture. PCA reduces the dimensionality of the data by focusing on the principal components that explain most of the variation. However, traits that are important for distinguishing species might not be those that contribute most to the overall variance, leading to potentially crucial information being discarded (Cadena et al. 2017). Our results show clear and deep separation into three groups corresponding to the three major geographic areas (Figure 5). On the PC1 axis, Texas populations were furthest from those found in the other two areas. On the PC2 axis, the Carolinas were most separated from the other two. There were no overlapping points between any of the clusters.

### 4.2 Species Delimitation and Taxonomy

**FIGURE 6:**
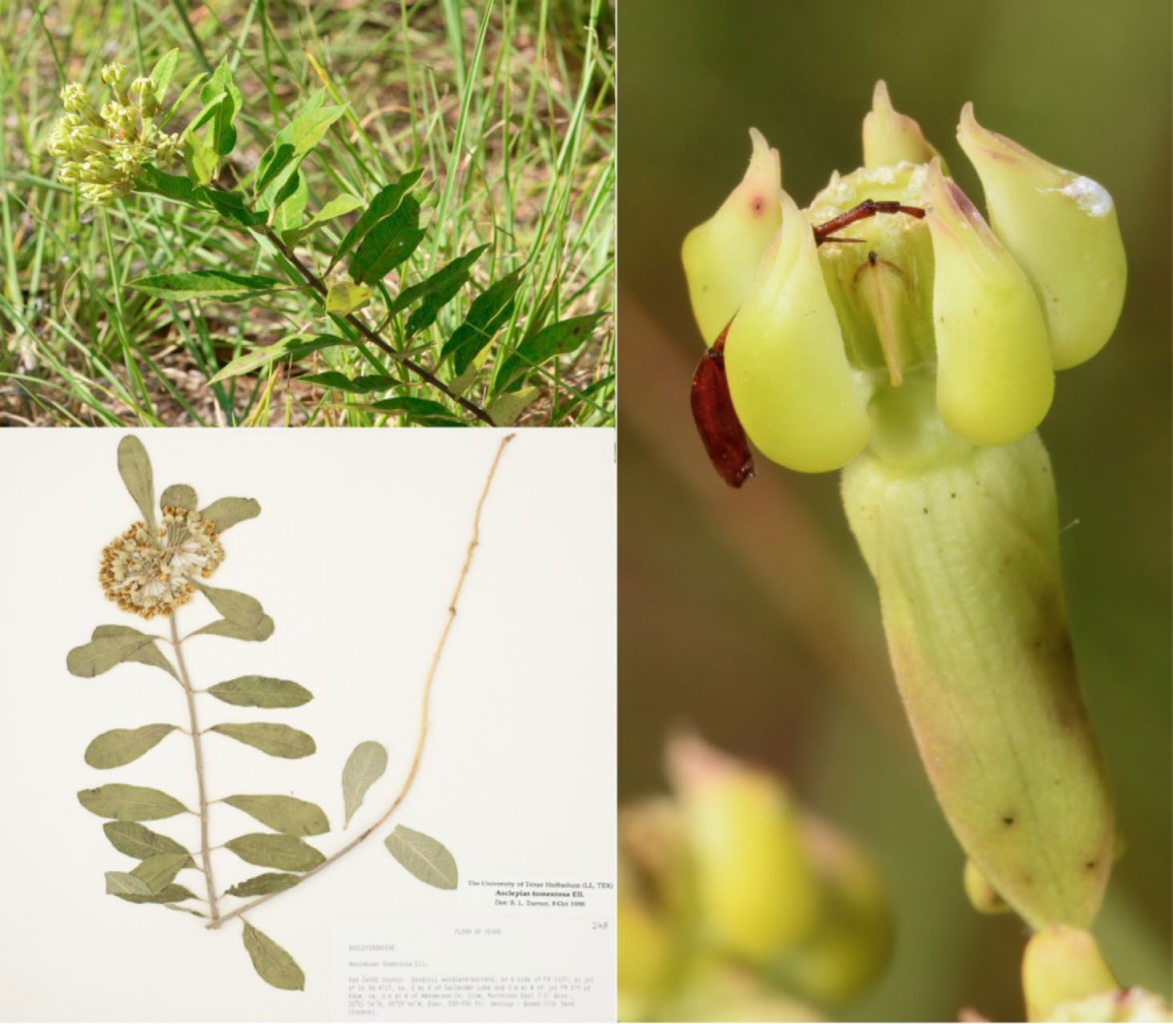
*Asclepias tonkawae* sp. nov. Upper left: Whole plant habitus, in situ (Anderson Co., Texas). Right: Detail of flower morphology, showing skirt‐like corolla appressed to the pedicel. Note beetle leg that is broken off between two horns. Lower left: Photograph of holotype (TEX).

We described *A. tonkawae*, sp. nov., as a distinct species because all genetic analyses demonstrated that it was substantially genetically distinct, and these patterns were also congruent with previously undocumented differences in floral morphology (Figures 2 and 6). Cryptic species may have subtle morphological or ecological differences that may go undetected by taxonomists for long periods of time since the original nominal species was described (e.g., Hebert et al. 2004; Gwiazdowski et al. 2011; Duran et al. 2019), although it is also possible that no detectable morphological differences may exist in otherwise genetically distinct species (Korshunova et al. 2019). In this case, the difference in floral morphology from *A. tomentosa* relates to the more strongly reflexed corolla, appressed to the pedicel in the former. No other clear, consistent differences in vegetative or floral characteristics appear between the two that the authors were able to observe. Given the greater depth of evolutionary divergence between *A. tonkawae* sp. nov. and sensu stricto *A. tomentosa* , it is not surprising that fixed morphological differences were present.

### 4.3 Distribution and Historical Biogeography

Both *A. tonkawae* and *A. tomentosa* are endemic to acidic eolian sands and appear to follow those sands as they spread through eolian or fluviatile mechanisms. Field observations in Florida by the third author indicate that *A. tomentosa* is associated with acidic sands and loams and is absent from alkaline or rocky soils. However, coastal populations demonstrate resistance to salt spray during major storm events. In addition to air dispersal of seeds, the species propagates by root breaking in shifting or eolian sands; propagation has been documented in natural conditions, as well as within sand roads or development sites where roots are broken by vehicular activity. It disperses readily across tombolo formations where eolian sand builds bridges to coastal islands. It does not appear to disperse well across water or wetlands to colonize barrier islands that have no history of connection to coastal sands. Florida appears to be the only place where populations occur on both ancient inland sands and on more recent coastal sands. Botanical fieldwork in the sandylands of Texas and the Carolinas has been extensive, but populations are only known from inland eolian sands and appear to be absent from Pleistocene barrier islands. In the Carolinas and Texas, carbonate or clay‐rich formations may block seaward dispersal.

*A. tomentosa* and *A. tonkawae* are sister species (Figure 3). A major question is where the ancestral species originated and how these disjunct sets of populations arrived where they are. Dispersal theories must be considered because milkweeds have wind‐blown seeds. If “sweepstakes” dispersal events occurred across 1,000 km via wind‐blown seeds, then we would expect more intermediate populations of *A. tomentosa* or *A. tonkawae* on sandylands in Louisiana, Mississippi, Alabama, and Georgia. Eocene sands are present in northern Louisiana. They have received attention from botanists who are familiar with *A. tomentosa* with negative results (MacRoberts and MacRoberts 1995).

As for a vicariance hypothesis, it can be informative to look at other obligate arenophiles with disjunct distributions in the same area. For example, the roach *Arenivaga floridensis* Caudell, 1918, is a Florida endemic with flightless females and over 20 endemic congeneric species in sandy areas of Texas and western North America (Deyrup 1990). The presence of this species in Florida could suggest the existence of a trans‐Mississippian sand bridge at some time in the past.

Finally, a submarine distributional hypothesis should be considered. During low stands of the Gulf of Mexico, the sea level was reduced by as much as 130 m. Vast areas of coastal land have been submerged as the sea level rose as recently as 10,000 years ago. Substrate maps of the Gulf of Mexico show large areas of sandy bottom that were terrestrial habitat at multiple times during the Pleistocene (Jenkins 2011). Schmidtling (2007) reported genetic evidence that longleaf pine ( *Pinus palustris* ) dispersed from Pleistocene refugia in Texas or Mexico eastward possibly, along the exposed continental shelf, to Florida through Virginia.

### 4.4 Conservation and Other Implications

Accurate species delimitation is critical for conservation efforts, as the rank of species is the unit most frequently protected by the Endangered Species Act of 1973. Critical re‐evaluation of traditional taxonomy in different species groups has revealed that species diversity has often been underestimated. In our study, we discovered a new species, *A. tonkawae* sp. nov., that is geographically restricted to a small section of eastern Texas (Figure 1). Given its limited distribution, it should be considered a high priority for conservation. *Asclepias tonkawae* sp. nov. only occurs in the loose sandy soils of the Carrizo and Sparta formations. We had a species conservation status assessment performed by Jason Singhurst of the Texas Parks and Wildlife Department using the NatureServe protocol (Master et al. 2012); the species would rank as G1 S1, or “critically imperiled,” globally and within the state of Texas, the only state where it occurs. Threats include extensive habitat conversion to non‐native pastures (coastal Bermuda and Bahia grass pastures); housing expanding in Tyler, Athens, Palestine, Quitman, Mineola, and other areas; conversion of habitat to monoculture pine; herbicide spraying to benefit grasses for grazing; natural fire regime highly reduced to non‐existent; and extensive land fragmentation.

Some populations have been found in protected government lands, such as Gus Engeling Wildlife Management Area (Anderson County) and Sabine National Forest (San Augustine County) and are likely to persist, provided that informed management practices are implemented.

One additional question is whether differences in floral morphology between *A. tonkawae* sp. nov. and *A. tomentosa* have implications for pollination. We have observed bumblebees (*Bombus* spp.) numerous times on inflorescences of sensu stricto *A. tomentosa* in both the Carolinas and Florida, as well as on *A. tonkawae* sp. nov. Given the different shape of the corolla, it is possible that other different pollinators are attracted to the floral morphology of *A. tonkawae* sp. nov., but this is currently unknown. More work is needed to characterize the pollinator community of both species.

### 4.5 Future Directions and Conclusions

We were unable to sample from the two known historic populations from Georgia Coffee County (southern Georgia) and Taylor County (western central Georgia), despite efforts to locate plants. One or both of these populations may no longer be extant, and both were isolated records that have not been observed since they were recorded. We hypothesize that the Coffee Co. population would be most closely related to the Florida clade, and the Taylor Co. population would fall within the Carolinas clade. The latter is likely historically connected to populations in the Carolinas given that it is in the same Fall Line Sandhills formation (Swezey et al. 2016). Further efforts to survey Georgia populations may provide insights as to the finer‐scale distribution of *A. tomentosa* , as well as help delineate potential cryptic species in that region, as suggested by our data. We welcome information regarding observations of *A. tomentosa* in Georgia.

This study uncovered cryptic species within nominal *A. tomentosa* using an integrative approach. Almost certainly, more cryptic species exist within the genus *Asclepias*, and our research highlights the potential for additional discovery. Given the already high diversity (~130 species in North America) and the fact that some nominal species exhibit large geographic ranges, named subspecies or forms, disjunct distributions, or ecological differences in parts of their ranges, more research is necessary to characterize species boundaries. A more accurate taxonomy and improved knowledge of species ranges can help conservation efforts and allow for an informed allocation of resources.

## 5 Taxonomic Treatment

Given the plurality of genomic results, we recognize the Texas populations of *A. tomentosa* as a separate species going forward. We found that the populations from Texas are supported as distinct based on reciprocal monophyly (Figure 3), considerable genetic divergence between Texas and other areas (*F*_ST_ = 0.296–0.357) (Table 1), separate clustering in the PCA (Figure 5), support from the Bayesian species delimitation model, and morphological differentiation. Aside from the reflexed corolla lobes, no other vegetative or floral morphological differences were observed between the Texas populations and the others. Although the majority of results also support the separation of Florida and Carolinas populations, at present, no apparent morphological differences exist, and the magnitude of divergence appears to be lower in all analyses; therefore, we believe the eastern populations warrant more study.

### 5.1 Asclepias tonkawae

**FIGURE 7:**
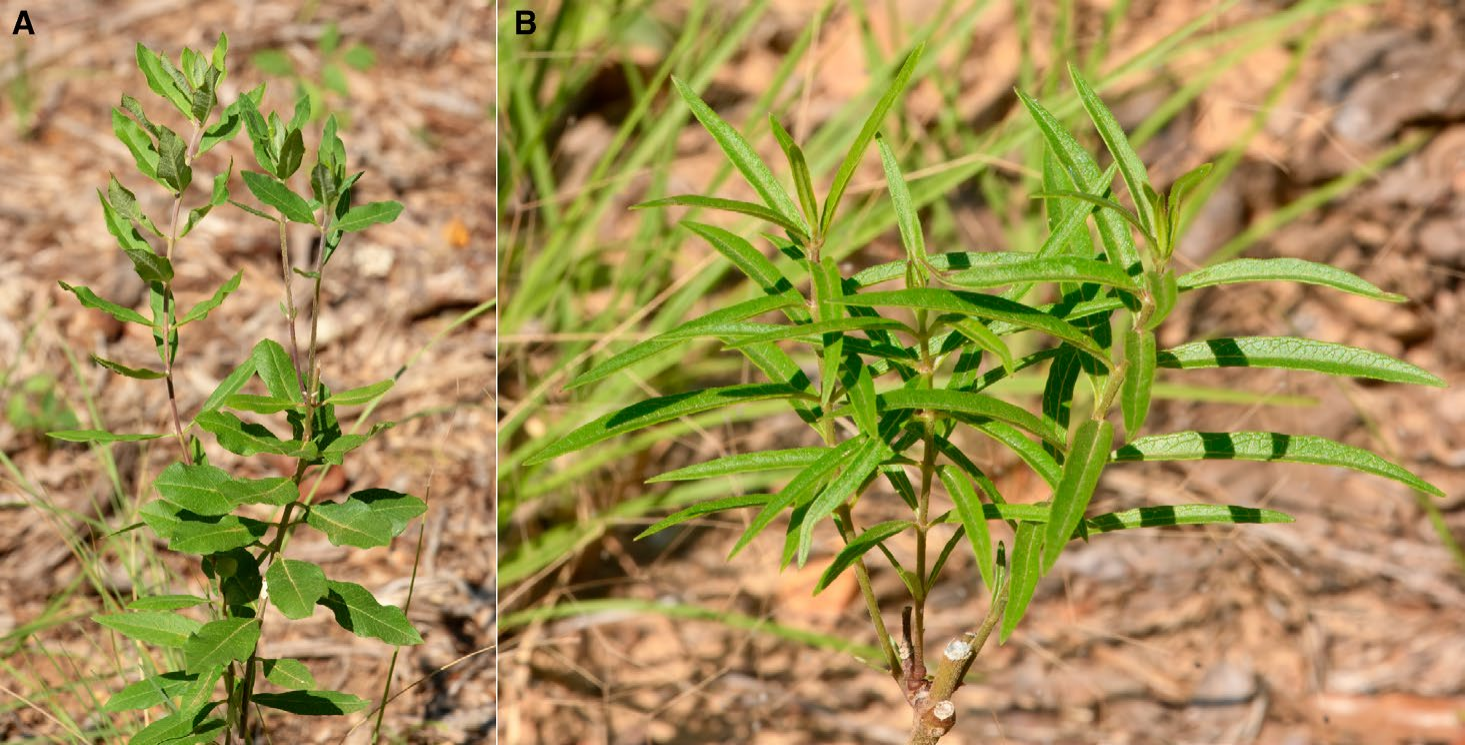
Variation in leaf morphology *Asclepias tonkawae* sp. nov. (Anderson Co., Texas). (A) Typical elliptical to obovate leaf morphology of mature plants, (B) Typical linear to lanceolate leaf morphology of young plants or new growth.

Duran, Ksepka, Davis, Godwin, Laroche, sp. Nov (Figures 6 and 7).

### 5.2 Differential Diagnosis

The new species resembles *A. tomentosa* in possessing stems that are never glabrous, green to white umbels sometimes suffused with pink or purple, pinnate‐reticulate veins in the leaves, erect follicles up to 15 cm, large deep‐seated tuberous roots, and a xerophytic habitat. The new species is differentiated from *A. tomentosa* by having the corolla strongly reflexed and appressed or nearly appressed to the pedicel.

### 5.3 Type

USA: TEXAS, Van Zandt County, on the N side of FR 2339, at the junction of Co Rd. 4717, ca. 2 mi E of Callender Lake and 3.6 mi W of the junction FM 279 at Edom, ca. 0.6 mi N of Henderson Co. line, Murchison East 7.5′ Quad., 32.365–95.66278. 19 August 1988. Collector: Steve L. Orzell and Edwin L. Bridge. Holotype University of Texas—Austin (TEX).

### 5.4 Description

Perennial forb, height to 55 cm. One to several stems from a tuberous rootstock, unbranched or branching after stem damage, puberulent to pubescent. Decussate leaf arrangement, simple, 8 cm long and 3 cm wide; blades variable, narrowly to broadly lanceolate, elliptic, oval to ovate, the apex acute or mucronate, the margin entire, crispate, or ciliate, the base cuneate, rounded, or weakly truncate; puberulent to pubescent on abaxial surface, sparsely pubescent on adaxial surface, with pinnately reticulate venation, petioles subsessile to 2 cm. Inflorescence of 1 terminal or several upper axillary umbels on peduncles 0.5–1.8 cm long; umbels of 12–25 flowers on puberulent to pubescent pedicels 2–5 cm long. Flowers 1.75–2 cm long from tip of corolla lobe to corona apex; calyx synsepalous, green, with 5 reflexed lobes 2–4 mm long and 2–3 mm wide; corolla sympetalous, green or suffused with dull pink to purple, the 5 strongly reflexed lobes 10–15 mm long and 5–7.5 mm wide; connate, columnar gynostegium of 6–7 mm long by 5 mm wide, comprised of 5 stamens fused to style apices, stamens bearing winged appendages to 2 mm, the cylindrical column composed of connate stamen filaments 2 mm long and 2 mm wide; corona comprised of 5 hoods arising from the base of the gynostegium, the erect hoods are dorsally flattened beyond midpoint, rounded basally, apex acute, 5–7 mm long, surpassing the gynostegium, green, bearing a narrow, adnate, reduced horn to 0.75 mm beyond hood apex. Fruits follicular, up to 15 cm long, 2 cm wide, fusiform, puberulent, and erect. The sap is a milky latex.

### 5.5 Phenology

Flowering from April to September, fruiting from May through October.

### 5.6 Etymology

The specific epithet is in honor of the Tonkawa Native American tribe whose historic and current lands overlap with the range of this new species. Furthermore, we support their history of conservation and land ethics practices. We give this species the common name of skirt milkweed owing to its skirt‐like corolla that is appressed to the pedicel.

### 5.7 Distribution and Habitat

Eastern Texas in Anderson, Franklin, Henderson, Houston, San Augustine, Shelby, Smith, Upshur, Van Zandt, and Wood Counties. Found in the Carrizo and Sparta sand formations. Habitat is xeric uplands within post oak savannas along sand ridges and ancient marine terraces dominated by *Quercus incana* and *Q. margarettae* . It should be noted that although the species occurs within intact natural communities, it also persists at ruderal sites, resprouting from roots.

### 5.8 Additional Specimens Examined (Paratypes)

USA: TEXAS, Franklin County, on the S side of FR 900, ca. 1.3 mi W of FM 115, 6.2 mi S of I‐30 on the S side of Mount Vernon, near two springs on the topo map, New Hope 7.5′; Quad. 33.07972–95.24694. 19 August 1988. Collector: Steve L. Orzell and Edwin L. Bridges. Sandhill woodlands and sand barrens, TEX00044127 (TEX); USA: TEXAS, Franklin County, Between “no trespassing” and wire fences, off the roadside on the S side of FM 900, ca. 0.2 mi W of County Rd. Southeast 3350, 1.2 mi E of SH 37, S of Mt. Vernon. 33.08057–95.24571 WGS84, 177 m, 02 August 2013. Collector: Lindsey Worcester, Mark Fishbein, Angela Rein, and Ben Haack, OKLA020044644 (OKLA); USA: TEXAS, Henderson, Moore Station Community, between Poyner and Brownsboro, off farm road 214. 12 September 1963. Collector: Donovan S. Correll and Helen B. Correll. In sandy soil in fields, LL00044124 (LL); USA: TEXAS, Marsh: Henderson County, 07 July 1924. Collector: Tharp, B.C., US1224416 (US); USA: TEXAS, Houston County, Grapeland. 08 June 1920. Collector: Tharp, B.C., US1104656 (US); USA: TEXAS, Houston County, Grapeland. 08 June 1920. Collector: Benjamin Carroll Tharp, TEX00044123 (TEX); USA: TEXAS, Houston County, Grapeland. 08 June 1920. Collector: Benjamin Carroll Tharp, SEU00005768 (SEU); USA: TEXAS, Smith County, Lindale. 09 June 1902. Collector: Reverchon, J. Sandy woods, US501006 (US); USA: TEXAS, Smith County, Lindale 09 June 1902. Collector: Reverchon, J., 101,055,374 (MO); USA: TEXAS, Smith County, Lindale 09 June 1902. 32.375035–95.269183 + −50,393 m. Collector: Reverchon, J. BRIT259884 (BRIT); USA: TEXAS, Upshur County, 10.2 miles west of Gilmer. 32.736277–94.941485 + −33,607 m. 01 July 1964. Collector: Lloyd H. Shinners. BRIT259885 (BRIT); USA: TEXAS, Wood County, Golden. 02 July 1926. Collector: E. McMullen. Fields and woods. TEX00044125 (TEX); USA: TEXAS, Wood County, on FM 3235, 0.25 mile southeast of Quitman Club Lake. 32.77433° N, −95.385637° W, WGS 1984. 17 August 2004. Collector: R.J. O'Kennon. BRIT232475 (BRIT).

## Author Contributions

**Daniel P. Duran:** conceptualization (equal), formal analysis (lead), investigation (lead), methodology (lead), project administration (lead), resources (equal), supervision (lead), visualization (lead), writing – original draft (lead). **Jason E. Ksepka:** conceptualization (equal), resources (equal), writing – original draft (supporting). **Scott A. Davis:** resources (equal), writing – original draft (supporting). **William Godwin:** funding acquisition (lead), resources (supporting), writing – original draft (supporting). **Robert A. S. Laroche:** data curation (lead), formal analysis (supporting), writing – review and editing (supporting).

## Disclosure

Benefits from this research include the new species description and distribution data that can be used in conservation efforts, as well as the broad benefits that accrue from the sharing of our data and results on public databases as described above.

## Conflicts of Interest

The authors declare no conflicts of interest.

## Supporting information


**Data S1:** GBS data.

**Figure S1:** Cross‐validation (CV) error used to determine optimal K for the population structure analyses (Figure 4).

**Table S1:** Collection localities for populations of the *Asclepias tomentosa* group sampled in this study.

**Table S2:** Measures of population genetic diversity within each of the three major clades identified during this study.

## Data Availability

The datasets generated during and/or analyzed during the current study are available in the following repository: GBS data was submitted to the NCBI Sequence Read Archive under accession numbers SAMN47323263–SAMN47323313. Links to all data are in Supporting Information.
