# Supplementary material for: Phylogenomics Reveals Deep Divergences and Cryptic Species Within a Rare Sand‐Dwelling Milkweed, Asclepias tomentosa Elliott
Source: Ecol Evol. 2025 Aug 8;15(8):e71942. doi: 10.1002/ece3.71942 (PMC12333076; doi:10.1002/ece3.71942)
Supplement: Supplementary file 1 — Data S1: GBS data. [file ECE3-15-e71942-s002.docx]

All GBS data from this study, deposited in the NCBI Sequence Read Archive:

<https://www.ncbi.nlm.nih.gov/biosample/47323263>
<https://www.ncbi.nlm.nih.gov/biosample/47323264>
<https://www.ncbi.nlm.nih.gov/biosample/47323265>
<https://www.ncbi.nlm.nih.gov/biosample/47323266>
<https://www.ncbi.nlm.nih.gov/biosample/47323267>
<https://www.ncbi.nlm.nih.gov/biosample/47323268>
<https://www.ncbi.nlm.nih.gov/biosample/47323269>
<https://www.ncbi.nlm.nih.gov/biosample/47323270>
<https://www.ncbi.nlm.nih.gov/biosample/47323271>
<https://www.ncbi.nlm.nih.gov/biosample/47323272>
<https://www.ncbi.nlm.nih.gov/biosample/47323273>
<https://www.ncbi.nlm.nih.gov/biosample/47323274>
<https://www.ncbi.nlm.nih.gov/biosample/47323275>
<https://www.ncbi.nlm.nih.gov/biosample/47323276>
<https://www.ncbi.nlm.nih.gov/biosample/47323277>
<https://www.ncbi.nlm.nih.gov/biosample/47323278>
<https://www.ncbi.nlm.nih.gov/biosample/47323279>
<https://www.ncbi.nlm.nih.gov/biosample/47323280>
<https://www.ncbi.nlm.nih.gov/biosample/47323281>
<https://www.ncbi.nlm.nih.gov/biosample/47323282>
<https://www.ncbi.nlm.nih.gov/biosample/47323283>
<https://www.ncbi.nlm.nih.gov/biosample/47323284>
<https://www.ncbi.nlm.nih.gov/biosample/47323285>
<https://www.ncbi.nlm.nih.gov/biosample/47323286>
<https://www.ncbi.nlm.nih.gov/biosample/47323287>
<https://www.ncbi.nlm.nih.gov/biosample/47323288>
<https://www.ncbi.nlm.nih.gov/biosample/47323289>
<https://www.ncbi.nlm.nih.gov/biosample/47323290>
<https://www.ncbi.nlm.nih.gov/biosample/47323291>
<https://www.ncbi.nlm.nih.gov/biosample/47323292>
<https://www.ncbi.nlm.nih.gov/biosample/47323293>
<https://www.ncbi.nlm.nih.gov/biosample/47323294>
<https://www.ncbi.nlm.nih.gov/biosample/47323295>
<https://www.ncbi.nlm.nih.gov/biosample/47323296>
<https://www.ncbi.nlm.nih.gov/biosample/47323297>
<https://www.ncbi.nlm.nih.gov/biosample/47323298>
<https://www.ncbi.nlm.nih.gov/biosample/47323299>
<https://www.ncbi.nlm.nih.gov/biosample/47323300>
<https://www.ncbi.nlm.nih.gov/biosample/47323301>
<https://www.ncbi.nlm.nih.gov/biosample/47323302>
<https://www.ncbi.nlm.nih.gov/biosample/47323303>
<https://www.ncbi.nlm.nih.gov/biosample/47323304>
<https://www.ncbi.nlm.nih.gov/biosample/47323305>
<https://www.ncbi.nlm.nih.gov/biosample/47323306>
<https://www.ncbi.nlm.nih.gov/biosample/47323307>
<https://www.ncbi.nlm.nih.gov/biosample/47323308>
<https://www.ncbi.nlm.nih.gov/biosample/47323309>
<https://www.ncbi.nlm.nih.gov/biosample/47323310>
<https://www.ncbi.nlm.nih.gov/biosample/47323311>
<https://www.ncbi.nlm.nih.gov/biosample/47323312>
<https://www.ncbi.nlm.nih.gov/biosample/47323313>
