# Supplementary figures and images for: Phylogenomics Reveals Deep Divergences and Cryptic Species Within a Rare Sand‐Dwelling Milkweed, Asclepias tomentosa Elliott
Source: Ecol Evol. 2025 Aug 8;15(8):e71942. doi: 10.1002/ece3.71942 (PMC12333076; doi:10.1002/ece3.71942)

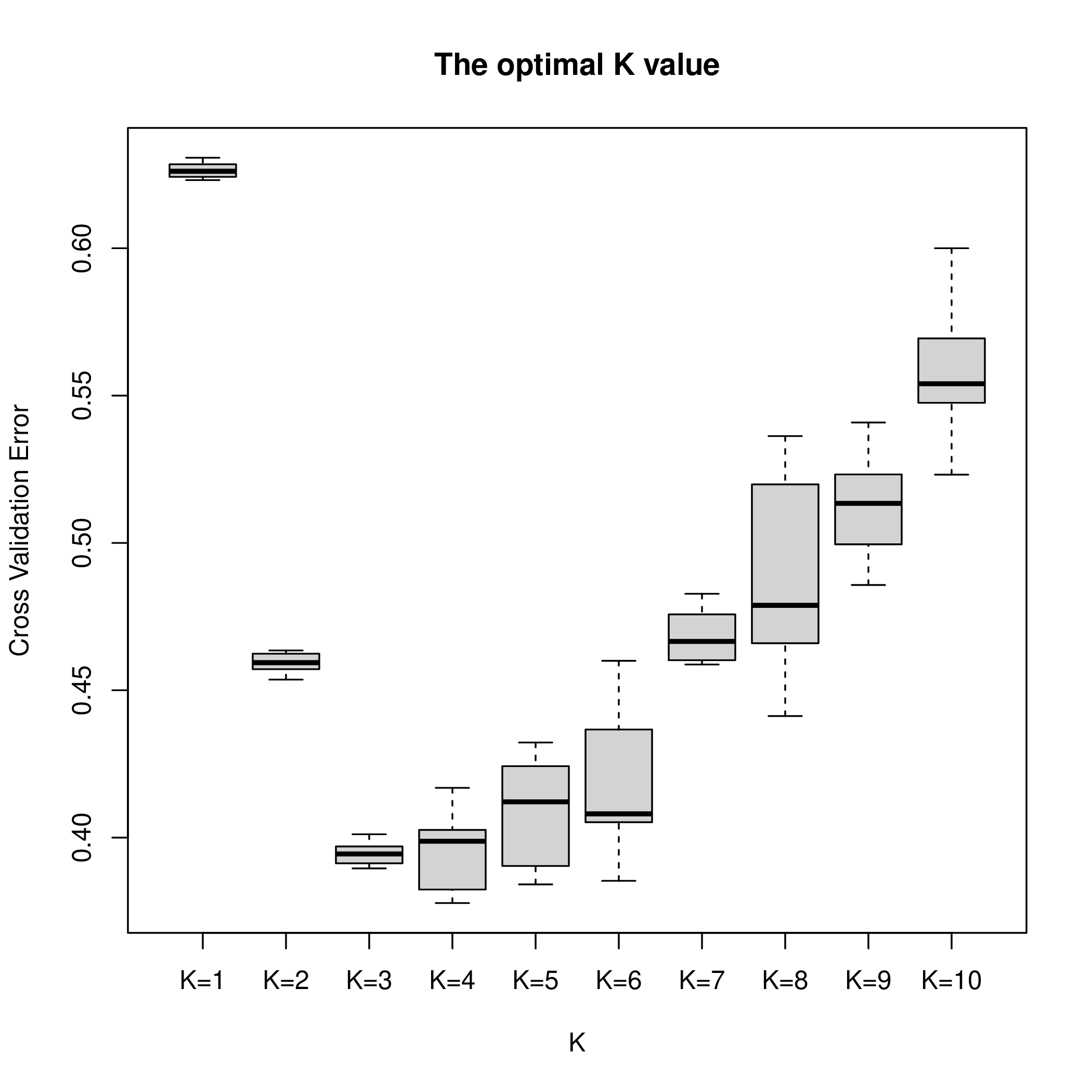

Supplement: Supplementary file 2 — Figure S1: Cross‐validation (CV) error used to determine optimal K for the population structure analyses (Figure 4). [file ECE3-15-e71942-s004.png]
